# Long-term evolution of antigen repertoires among carried meningococci

**DOI:** 10.1098/rspb.2009.2033

**Published:** 2010-02-03

**Authors:** Caroline O. Buckee, Sunetra Gupta, Paula Kriz, Martin C. J. Maiden, Keith A. Jolley

**Affiliations:** 1 Department of Zoology, University of Oxford, South Parks Road, Oxford OX1 3PS, UK; 2 Santa Fe Institute, 1399 Hyde Park Road, Santa Fe, NM 87501, USA; 3 National Reference Laboratory for Meningococcal Infections, National Institute of Public Health, Srobarova 48, Prague, Czech Republic

**Keywords:** *Neisseria meningitidis*, bacterial population structure, network

## Abstract

Most studies of bacterial pathogen populations have been based on isolates collected from individuals with disease, or their contacts, over short time periods. For commensal organisms that occasionally cause disease, such as *Neisseria meningitidis*, however, the analysis of isolates from long-term asymptomatic carriage is necessary to elucidate their evolution and population structure. Here, we use mathematical models to analyse the structuring and dynamics of three vaccine-candidate antigens among carried meningococcal isolates collected over nearly 30 years in the Czech Republic. The data indicate that stable combinations of antigenic alleles were maintained over this time period despite evidence for high rates of recombination, consistent with theoretical models in which strong immune selection can maintain non-overlapping combinations of antigenic determinants in the presence of recombination. We contrast this antigenic structure with the overlapping but relatively stable combinations of the housekeeping genes observed among the same isolates, and use a novel network approach to visualize these relationships.

## 1. Introduction

*Neisseria meningitidis*, the meningococcus, is a genetically and antigenically diverse bacterial cause of meningitis and septicaemia, which is responsible for appreciable levels of morbidity and mortality worldwide (Harrison *et al*. 2009). A comprehensive vaccine is yet to be developed against this encapsulated organism (Jodar *et al*. 2002), and studies of its epidemiology have demonstrated that an understanding of the meningococcal evolution and population structure, particularly the long-term stability of associations between different antigens, is essential for the design and implementation of comprehensive vaccines (Caugant & Maiden 2009).

The polysaccharide capsule is an important vaccine antigen, as virulent meningococci are almost always encapsulated, and capsule variants define the 13 meningococcal serogroups (A, B, C, etc.). Meningococci from asymptomatic carriage may or may not express a capsule, either because the capsule genes are absent or because they are downregulated (Weber *et al*. 2006). Subcapsular outer membrane proteins (OMPs) are used to further characterize meningococci and are also candidate vaccine components. One of these, the PorA protein, defines meningococcal serosubtype and is a component of strain-specific vaccines (Bjune *et al*. 1991; Sierra *et al*. 1991; O'Hallahan *et al*. 2004; Hosking *et al*. 2007; Kelly *et al*. 2009). It has two antigenically variable regions (VR1 and VR2; McGuinness *et al*. 1993), which show great diversity on a population level, with 196 VR1 and 534 VR2 peptide variants described at the time of writing (Russell *et al*. 2004). The PorA variable regions have been shown to be structured into non-overlapping combinations (Gupta *et al*. 1996; Buckee *et al*. 2008), consistent with theoretical predictions that strong immune selection may polarize the pathogen population into discrete antigenic types (Gupta *et al*. 1996). Here we extend this analysis to include another promising vaccine candidate, the OMP FetA, which has a single diverse highly immunogenic variable region (Smith *et al*. 1993; Jolley *et al*. 2005). To date there have been no long-term studies of the structure of FetA with respect to the PorA variable regions or housekeeping genes, although the utility of OMP-based vaccines based on these antigens will rely on the structure of diversity at these loci, the stability of different antigenic variants in the meningococcal population over time and their association with virulence factors.

We use a novel network approach to analyse the population structure of the PorA variable regions and FetA within a study of carriage isolates in the Czech Republic spanning 27 years, as well as their relationship with genetic lineages defined by multilocus sequence typing (MLST). We compare the data to a stochastic model of pathogen evolution to confirm that the structure of the PorA variable regions is both stable and non-overlapping, indicating that strong immune selection is acting on these regions. We show that the FetA variable region shows much less stable structure, although on short time scales strong associations between FetA and PorA variable regions are apparent. We contrast these structures with the relationships between housekeeping loci, which exhibit considerable structuring but no evidence of immune selection.

## 2. Material and methods

### (a) Bacterial isolates and sequencing

A total of 1054 meningococci were isolated from throat swabs between 1971 and 1983, and from 1992 to 1997 in the Czech Republic, as described previously (Jolley *et al*. 2000; Buckee *et al*. 2008). All the samples originated from healthy individuals with no known contact to cases of invasive disease. Collection of throat swabs and microbiology, as well as nucleotide sequencing of isolates, is described in Buckee *et al*. (2008). PorA and FetA variable region sequences were determined using published methods (Thompson *et al*. 2003; Russell *et al*. 2004).

### (b) Measuring the stability of associations between alleles

A standardized measure of two-locus associations, D* (Hedrick & Thomson 1986), was used to analyse the stability of different types of allelic associations across the 27-year span of the dataset (for derivation of D*, see the electronic supplementary material). Unlike other linkage statistics that deal with multiple loci and limited allelic diversity, this measure compares observed and expected frequencies of multiple alleles at two loci and tests for an association between them (expected frequencies are calculated according to assumptions of neutrality for a haploid genome). A high D* score is indicative of highly stable linkage, whereas a low score indicates minimal linkage between loci, or fluctuating associations over time in this context, since changes in association over time between alleles at two loci will lower the overall association.

### (c) Measuring overlap

Several studies have shown that strains may polarize in strain space under strong immune selection (Gog & Grenfell 2002; Gog & Swinton 2002; Gomes *et al*. 2002), producing a structured pathogen population. Lineages defined by genes that are not under immune selection may also show strong structuring, however. Here, we are explicitly testing the hypothesis that immune selection will generate non-overlapping combinations of alleles and a pathogen population characterized by discrete strains (Gupta *et al*. 1996), as distinct from structure caused by processes other than direct immune selection. To that end, we introduce a simple metric, f*, for quantifying the extent of the overlap between alleles at two loci. A stochastic individual-based model of pathogen evolution was used to generate hypothetical distributions of allelic associations and test the f* statistic (see the electronic supplementary material for derivation of f* and details of the model). In this model, strain types were defined by three loci, two antigenic and one housekeeping, each with five alleles (thus, strain space becomes a 5 × 5 × 5 matrix of allelic associations). The two antigenic loci determined host immunity, with hosts gaining a degree of protection against strain types sharing alleles with a previously encountered strain type dependent on *γ*, the level of cross-protection. More details are given in Buckee *et al*. (2004, 2008). The housekeeping gene product generated no immune response; however, it was subject to the same rates of mutation and recombination as the antigenic genes. Simulations were run with different levels of cross-immunity until they had reached equilibrium, and the frequencies of allele combinations in the 5 × 5 × 5 matrix were then analysed (see the electronic supplementary material, fig. S2). A clear nonlinear decrease in overlap (the inverse of f*) between combinations of antigenic variants is seen as immunological cross-protection (*γ*) increases. By contrast, there is no effect on the associations between housekeeping genes and antigenic variants.

### (d) Visualizing relationships between isolates

In order to visualize the differences between the structural and temporal relationships of the antigens versus the housekeeping genes, a network technique was used where each isolate is represented as a node in a network, with edges between nodes representing shared alleles between isolates. In this case, the same number of housekeeping gene loci as antigenic determinants (three: two PorA variable regions and one FetA variable region) were used for each comparison, to avoid bias due to the number of genes used in the analysis. Note that unlike the visualizations generated by programs such as eBurst (Feil *et al*. 2004), no attempt was made to infer the evolutionary relationships between isolates, since antigenic loci are under strong immune selection and recombination rates are high. In the extreme hypothetical case with completely non-overlapping combinations, the large stable nodes would share no links with each other. The network visualization indicates the extent to which recombination disrupts the structure of antigen combinations, therefore, and highlights the differences in the allelic relationships between the housekeeping loci and antigens of the same isolates. Although stable combinations of alleles may exist among genes not under immune selection due to descent, there is no reason why they will not share alleles at certain loci; thus, networks of housekeeping genes are expected to show less differentiation between dominant clusters of allele combinations.

Various standard metrics of network structure were measured to determine how the relationships between antigenic and housekeeping loci vary, including the density of the network, the clustering coefficient, the betweenness centrality of the nodes (which measures the relative importance of each node in connecting others to the network) and the degree of distribution (how many links, or edges, each node has). The derivation of these metrics can be found in the electronic supplementary material. Each reflects the structure of the network and the extent to which the nodes cluster into distinct groups, indicative of immune selection.

## 3. Results

### (a) Stability of alleles over time

For each antigenic and housekeeping locus sequenced, very few variants were of intermediate longevity, the dataset being comprised mainly of a large number of low-frequency, short-lived variants appearing in the data only once, and a smaller number of dominant variants that lasted for more than 20 years. For example, out of a total of 82 PorA VR2 variants, 35 (43%) were observed only once while 28 (34%) were seen over at least 20 years, with the latter representing 894 (85%) of the isolates. With combinations of variants, the results were more pronounced. For example, out of 352 PorA VR2∶FetA VR combinations, 212 (60%) were observed once but accounted for only 21 per cent of the isolates, whereas 40 (11%) were seen over 20 years, comprising 41 per cent of the isolates. The housekeeping genes had a mean of 59 unique alleles per locus, of which 24 (41%) were seen only once with just 17 (29%) observed over 20 years, yet accounting for 907 (86%) of the isolates. The long-term stability of alleles among these isolates has previously been reported (Buckee *et al*. 2008).

### (b) Stability of associations between different types of genes

**Figure 1.**
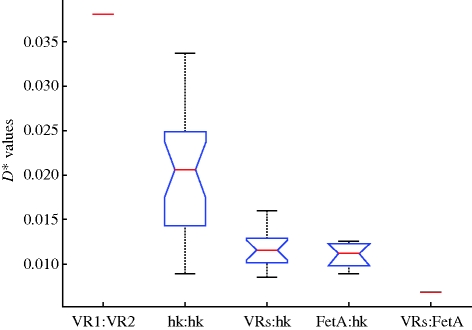
The association between different pairs of loci over 26 years in the Czech Republic. D* values for each pair of loci was calculated. The boxplots show the median (red line), lower and upper quartiles (blue box) and the extent of the range (whiskers) for those loci that involved more than one comparison; for example, the calculation of D* for each housekeeping gene compared with every other housekeeping gene led to a total of 21 D* scores. For the PorA VR associations, only a single metric was computed between VR1 and VR2 (hence the single line shown on the plot). For the FetA∶PorA VR comparisons, two calculations were made: FetA versus PorA VR1 and FetA versus PorA VR2 (shown by the blue box). ‘VR’ refers to the variable regions of PorA, ‘hk’ refers to housekeeping genes, and ‘FetA’ represents the variable region of FetA. The concatenation of these indicates the comparison made. The PorA VR and housekeeping gene associations were significantly higher than the other two-locus comparisons (*F* = 108.15, *p* = 10^−11^), and the PorA VR associations were significantly different from the housekeeping gene associations, but only marginally (*F* = 6.32, *p* = 0.0189).

The linkage statistic D* (Hedrick & Thomson 1986) was used to test the stability of associations between different loci over the 27 years. PorA variable regions showed relatively high D* values, indicative of strong, stable associations between the two regions over time. However, both VR1∶FetA and VR2∶FetA comparisons had a low D* value in comparison to the PorA variable regions, indicating that linkage between these loci was not maintained to the same extent (figure 1). Combinations of VR2∶FetA changed over the period of the study, with particular combinations changing in dominance. For example, 2-2∶F4-3, representative of the ST-549 complex, was seen extensively in 1972–1973 (42 isolates, 18%), but then was not observed again, whereas 3 : F3-6 was predominant in 1974–1975 (24 isolates, 11%). The emergence of the ST-11 complex in 1993–1994 was marked by the 2∶F3-6 combination (35 isolates, 12%), which was not observed previously. The housekeeping gene versus housekeeping gene linkage was high over time (figure 1), significantly higher than the association between housekeeping and antigenic genes. The lower linkage of the PorA VRs and FetA with the housekeeping genes suggests that stable lineages defined by housekeeping genes have a fluctuating association with particular antigenic determinants, as is also evident from a qualitative inspection of the data and previous analyses (Buckee *et al*. 2008). Note that we use D* here as a relative rather than absolute measure of association between two loci.

### (c) Non-overlapping structure

**Figure 2.**
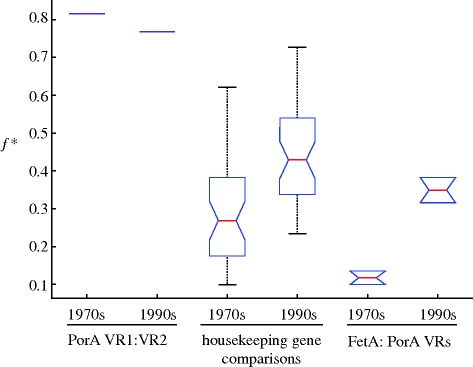
The distribution of f* scores from the data split into the 1970s (1971–1983) and the 1990s (1992–1997). The number of calculations for each type of two-locus comparison was the same as for the D* scores. The housekeeping gene comparisons represent all three types of comparison: housekeeping gene versus housekeeping gene or PorA VRs or FetA scores (these three groups are not significantly different from each other, as expected).

Each locus was compared with every other locus in a two-way comparison to determine the extent of allelic overlap between them for the two time periods (figure 2), and the f* metrics for these comparisons were analysed with respect to the model output (model results are shown in the electronic supplementary material). The combinations of PorA VRs observed were not only stable over time, despite fluctuations in frequency, but also showed discrete non-overlapping structure with an f* metric comparable to the antigenic loci in the model under assumptions of strong cross-immunity. In similar accordance with the model, the housekeeping genes, which are not under immune selection, did not show the distinctive non-overlapping structure observed between the two PorA variable regions, either when compared with other housekeeping loci or to antigenic loci.

The non-overlapping structure observed among PorA VR1∶VR2 combinations was most pronounced when the dataset was split into two time periods; however, the non-overlapping structure remained strong even when the entire time period was considered (f* = 0.76). The stability of these dominant combinations was consistent with the model of immune selection described in §1, in which selection is strong enough to stably maintain antigenic combinations. The f* values obtained when PorA VRs were compared with the FetA locus could not be distinguished from the housekeeping gene comparisons, however, and during the 1970s actually exhibited a lower f* score than the housekeeping gene comparisons. For example, between 1971 and 1975 FetA allele 1-7, the most prevalent allele in the dataset, is associated at relatively high frequencies with PorA VR1 alleles 7-1 (7 isolates), 12-1 (7 isolates), 18 (13 isolates) and 22 (15 isolates), substantially reducing the f* score. When the 2 years with the largest numbers of isolates were examined independently, however (1972 and 1993), the f* metric for FetA with respect to PorA VR2 rose substantially to 0.585 and 0.618, respectively—significantly higher than the housekeeping gene comparisons from this period. The short-term nature of the associations between FetA and PorA is also emphasized by the fact that the 1990s comparison, which reflects a much shorter time scale (1992–1997), is significantly higher than the 1970s comparison.

**Figure 3.**
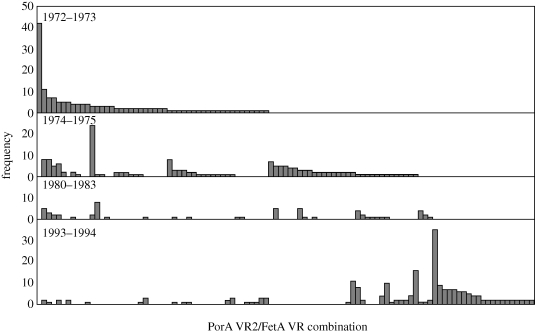
The longevity and prevalence of PorA VR2∶FetA VR variant combinations in the dataset. Each bar represents a particular combination of variants that are seen at least twice within the dataset and their order is determined by their frequencies in successive time periods.

Distinct structuring of associations for some of the most prevalent FetA variants with respect to the PorA variants was apparent when individual years were examined (figure 3). Each FetA allele was primarily associated with one combination of PorA VR1∶VR2, although this changed over time. For example, the FetA allele F3-6 was associated with PorA VR combination 18-1∶3 in the 1970s, and with 10-1∶5-1 in the 1990s. The relationship was not reciprocated, however, with one VR1∶VR2 allele combination being associated with a number of FetA alleles at any point in time, resulting in a low-scoring f* value. Thus, the FetA variable region showed strong associations with the PorA variable regions, but on a relatively short time scale.

### (d) Network visualizations

**Figure 4.**
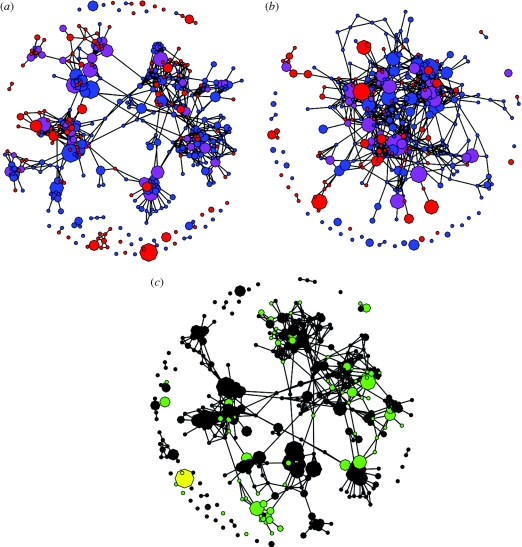
(*a*) Network visualization of the relationships between isolates for the three antigenic determinants, PorA VR1, PorA VR2 and FetA, showing the time period of combinations: the 1970s (blue), the 1990s (red) and both time periods (purple). (*b*) Three housekeeping genes, in this case *gdh*, *pdhC* and *pgm*, coloured in the same way as (*a*). (*c*) The antigen network, coloured according to two different clonal complexes—ST-41/44 (green) and ST-11 (yellow)—showing the different types of association between the antigens and housekeeping genes in the data.

Compared with the housekeeping gene networks, the antigen network showed approximately the same density of edges per node, and equivalent numbers of long-lived combinations (figure 4*a*,*b*). Highly significant differences were observed in the level of clustering, or transitivity (*F* = 86, *p* = 10^−11^), however, with the antigens showing strong clusters separated from each other. This is also reflected in the significantly higher mean betweenness centrality per node (*F* = 42, *p* = 10^−7^) and degree (*F* = 22.3, *p* = 10^−5^), indicating that clusters of dominant nodes are loosely linked to the rest of the network, or show little overlap with other clusters compared with the housekeeping gene networks. Furthermore, there was a significant positive correlation in the antigen network between vertex size (the number of isolates with a particular combination of alleles) and degree; in other words, the stable combinations continually recombine, causing large numbers of links within clusters, but recombinants between clusters are transient.

The associations of antigen variant combinations and clonal complexes were highly variable, as exemplified by two major lineages, the ST-41/44 and ST-11 clonal complexes (figure 4*c*), with some strain types being associated with a highly limited set of PorA∶FetA combinations (ST-11), and others being associated with a broad range of different combinations that change with time (ST-41/44). These two examples illustrate how the range of epidemiological patterns exhibited by different clonal complexes affects their relationships with antigenic determinants and highlight the difficulties inherent to developing OMV-based vaccines against hypervirulent lineages. In this case, it seems probable that ST-11 may be easier to target in the short term than ST-41/44.

## 4. Discussion

The absence of long-term carriage data for *N. meningitidis* and other primarily commensal bacteria has hindered our understanding of their population structures. In this study, we have presented the long-term antigenic structuring of carriage isolates collected in one country over almost three decades. The longevity of alleles and allelic combinations, with a large number of low-frequency, short-lived alleles and a small number of dominant alleles lasting more than 20 years, is consistent with previous MLST studies of meningococcal carriage isolates (Jolley *et al*. 2000; Urwin *et al*. 2004; Yazdankhah *et al*. 2004; Jolley *et al*. 2005). It suggests that certain alleles and combinations of antigenic variants are extremely stable over time, with no antigenic drift occurring, although recombination continuously generates large numbers of transient variants.

The stability of associations within the housekeeping genes and the non-overlapping structure of the PorA epitopes, with a cycling of pre-existing antigenic types, is consistent with previous studies concluding that the epidemic clone model cannot explain the population structure of these *N. meningitidis* loci (Urwin *et al*. 2004; Buckee *et al*. 2008). Instead, stable genetic lineages appear to show a fluctuating association, with stable combinations of antigenic determinants. This suggests that different selection processes are occurring independently at housekeeping and antigenic loci, facilitated by high rates of recombination. Urwin *et al*. (2004) demonstrated the clustering of antigenic types with hyperinvasive clonal complexes, but of these only the ST-11 and ST-41/44 complexes appear within this dataset at a frequency sufficient for antigenic associations to be apparent. As observed in our antigen networks, the ST-11 complex is the least diverse antigenically (almost exclusively 2∶F3-6), while the ST-41/44 complex is more diverse at both the housekeeping and antigen level, although some temporal clustering of antigen types is apparent, in concordance with Urwin *et al*. (2004). Antigenic shift has been noted in *N. meningitidis* populations in North America (Harrison *et al*. 2006; Tsang *et al*. 2006), where amino acid substitutions occurred simultaneously in the PorA VR1 and VR2, PorB and FetA VR antigens. These shifts coincided with an increase in meningococcal disease incidence. Prior to ST-11 emerging in the Czech Republic in 1993 (Krizova *et al*. 1997), identified by a characteristic antigen repertoire of serogroup C, PorA 5,2; FetA F3-6, and causing a marked increase in disease, there was no incidence of either of these PorA VRs among serogroup C strains. Similarly, in our dataset, the FetA F3-6 was only associated twice with serogroup C prior to this, in 1975 and 1978. There were no other strains circulating during, or immediately prior to, 1993 that expressed any of these antigen types, so it seems probable that the emergence was assisted by a lack of immunity to this particular strain among the human population.

The structuring of the PorA variable regions is consistent with models in which strong immune selection leads to stable, dominant strain types expressing non-overlapping combinations of alleles at antigenic loci (Gupta *et al*. 1996). The association between PorA and FetA appears to be less stable over time, although strong associations exist at any point in time. It is possible that FetA is less immunogenic than the PorA variable regions, producing an oscillatory dynamic corresponding to moderately high levels of cross-immunity within the model framework (Gupta *et al*. 1996), although more data are needed to test this hypothesis. Despite this dynamic association, however, the antigenic network including the FetA variable region showed a strong clustered structure, which we propose is due to immune selection. Although it is also a formal possibility that the low level of overlap of PorA VR epitopes is caused by their proximity on the same gene, we contend that the overall diversity of associations observed and the frequent generation of recombinant combinations makes this unlikely. More large studies of carriage isolates over at least a few years are needed to verify whether the oscillatory behaviour between FetA and PorA is consistently observed, since the number of single variants can swamp the data for shorter time scales.

We have introduced a simple metric for quantifying the extent of allelic overlap between loci, and a straightforward way to visualize relationships between isolates. Although several theoretical studies have shown that strong immune selection can polarize pathogen strains within strain space (Gog & Grenfell 2002; Gog & Swinton 2002; Gomes *et al*. 2002), these have not explicitly predicted non-overlapping combinations of alleles among discrete antigenic types. Our f* measure can test explicitly for this type of structuring, and distinguishes well between the housekeeping gene comparisons—which exhibit strong but overlapping lineage structure—and the PorA VR epitopes. For diverse, immunodominant antigens, it is a useful indicator of the extent of this non-overlapping structure and can provide a signal of high levels of cross-immunity, unrestricted by other constraints. For antigens that are known to be immunogenic but do not show distinct non-overlapping structure, either cross-immunity is relatively lower, causing oscillatory associations between loci, in which case the f* score should increase when an appropriate time scale is analysed, or there is some constraint upon the antigen in its response to immune selection. By visualizing the relationships between isolates as a network, we introduce a simple and transparent method for assessing the structural differences between antigens and housekeeping genes. Since the edges of the network reflect overlap between allelic combinations, this technique is a simple way to assess the extent of non-overlapping structure that we expect when immune selection is strong.

The long-term stability of a subset of dominant antigenic determinants in these data, in spite of fluctuations in their associations, suggests that the overall diversity of the *N. meningitidis* population may not be an insurmountable problem for vaccine design. Although the combination of alleles at the PorA and FetA loci may change over time, even within the relatively less diverse outbreak strains, there appear to be particular dominant variants that are stable through time in spite of recombination. The large amount of diversity of the pathogen population at any point in time is therefore largely due to transient recombinants generated within hosts and does not necessarily prohibit the development of an effective OMP-based vaccine.
